# Constraint lines and performance envelopes in behavioral physiology: the case of the aerobic dive limit

**DOI:** 10.3389/fphys.2012.00381

**Published:** 2012-09-26

**Authors:** Markus Horning

**Affiliations:** Department of Fisheries and Wildlife, Marine Mammal Institute, Oregon State UniversityNewport, OR, USA

**Keywords:** aerobic dive limit, dive response, oxygen debt, constraint line, distribution boundary, performance envelope, edge effect, quantile regression

## Abstract

Constraint lines—the boundaries that delimit point clouds in bivariate scattergrams—have been applied in macro-ecology to quantify the effects of limiting factors on response variables, but have not been applied to the behavioral performance and physiological ecology of individual vertebrates. I propose that behavioral scattergrams of air-breathing, diving vertebrates contain informative edges that convey insights into physiological constraints that shape the performance envelopes of divers. In the classic example of repeated cycles of apnea and eupnea in diving, air-breathing vertebrates, the need to balance oxygen consumption, and intake should differentially constrain recovery for dives within or exceeding the aerobic dive limit (ADL). However, the bulk of variance observed in recovery versus dive duration scattergrams originates from undetermined behavioral variables, and deviations from overall stasis may become increasingly apparent at progressively smaller scales of observation. As shown on dive records from 79 Galápagos fur seals, the selection of appropriate time scales of integration yields two distinct recovery boundaries for dive series within and beyond the estimated ADL. An analysis of the corresponding constraint lines is independent of central tendencies in data and avoids violating parametric assumptions for large data sets where variables of interest account for only a small portion of observed variance. I hypothesize that the intercept between these constraint lines represents the effective ADL, and present physiological and ecological considerations to support this hypothesis.

## Introduction

Physiology investigates mechanisms for maintaining homeostasis over a range of temporal and spatial scales (Costa and Sinervo, 2004). The scale at which an organism and the surrounding environment are sampled is particularly important: within the scope of behavioral and physiological plasticity, deviations from stasis may increasingly occur at progressively smaller spatial and temporal scales. To reduce the impact of variation secondary to plasticity, physiological experiments in the laboratory often attempt to create steady state conditions to reduce residual variation and to analyze constraints and limiting factors (Costa and Sinervo, 2004). However, physiological data collected in the field rarely reflect steady state conditions and significant deviations from homeostasis are readily observed. A classic example is the repeated cycle of apnea and eupnea in diving, air-breathing vertebrates. On a larger scale, oxygen consumption and intake need to be balanced. On a small scale, significant deviations occur during every dive as animals deplete stored oxygen to differing degrees and may even increase oxygen debt by temporarily switching to less efficient anaerobic metabolism (Kooyman et al., 1981; Kooyman, 1989). This subsequently requires additional oxygen to remove metabolites that would otherwise threaten homeostasis. The need to balance the use of oxygen stored and used in different tissue may be further modulated through the temporal partitioning of some activities that are obligate only on a longer scale, such as digestion, detoxification, mobilization of stored energy, or even the replenishment of oxygen stores (Davis et al., 1983; Kooyman, 1989; Ponganis et al., 1993).

### The aerobic dive limit

The aerobic dive limit (ADL) was originally defined by G. L. Kooyman as the dive duration associated with the onset of blood lactate accumulation, presumably resulting from anaerobic energy production following oxygen depletion in some tissue (Kooyman et al., 1980). Available oxygen stores and consumption rates are key determinants of the onset of lactate accumulation, in conjunction with multiple fine scale regulatory adjustments commonly referred to as the “dive response” (Kooyman et al., 1981; Butler and Jones, 1997; Kooyman and Ponganis, 1998; Davis and Kanatous, 1999). As a result of the central and integrative positioning within energy metabolism, the ADL has been proposed as a useful concept in comparative diving physiology in a number of variants that include calculated and behavioral threshold estimates if empirical lactate measurements are impossible (Kooyman, 1989; Kooyman and Ponganis, 1998; Butler, 2006; Ponganis et al., 2010). The ADL may be considered an intrinsic trait subject to natural selection (Butler and Jones, 1997; Davis et al., 2004), and has been used for interspecific comparisons (Kooyman, 1989; Boyd and Croxall, 1996; Kooyman and Ponganis, 1998) and intraspecific studies on ecology, ontogeny and aging (Kooyman et al., 1983; Gentry and Kooyman, 1986; Thorson and Le Boeuf, 1994; Burns and Castellini, 1996; Horning and Trillmich, 1997a; Ponganis et al., 1997; Kooyman and Ponganis, 1998; Watanuki and Burger, 1999; Pitcher et al., 2005; Richmond et al., 2006; Weise and Costa, 2007; Cook et al., 2008; Hindle and Horning, 2010; Hindle et al., 2011; Shero et al., 2012). These studies suggest that some species and age classes remain predominantly aerobic for most dives, while others—birds in particular—frequently dive well in excess of the estimated ADL. However, the extent to which the ADL represents an actual threshold that constrains foraging behavior, energy acquisition, and ultimately individual fitness remains unclear (Davis et al., 2004; Ponganis et al., 2010, 2011).

**Figure 1:**
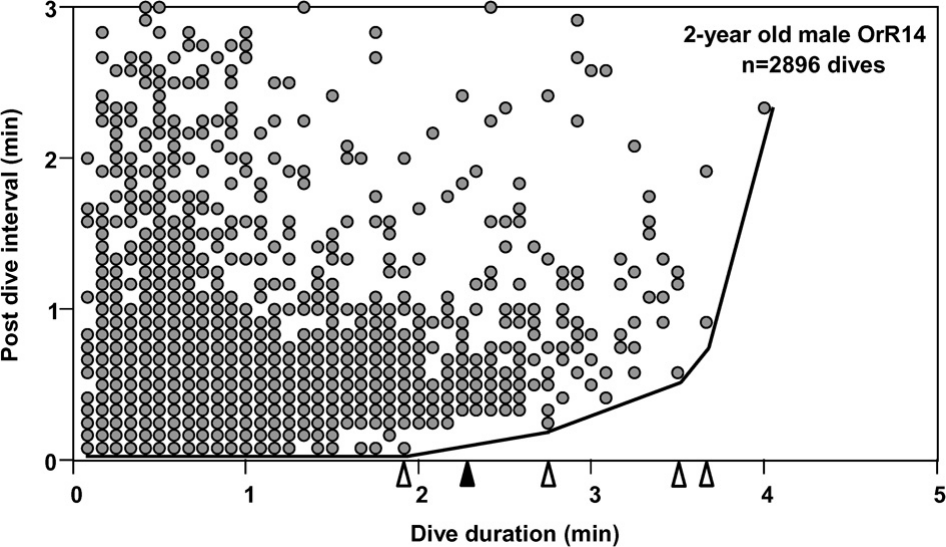
**Post-dive recovery intervals are plotted as a function of dive duration, for 2-year old male Galápagos fur seal OrR14.** Data pairs from 2896 dives recorded during 18 deployment days are depicted. Due to sampling at a discrete rate of 5 s, many data points are plotted on top of each other. Open triangles identify four possible inflection points in a bottom contour line (see text). The solid black triangle identifies the dive duration associated with the Surface Time Inflection point (see text).

To understand the limiting effects of intrinsic traits such as the ADL on individual behavior and ultimately fitness, we may analyze variance in behavioral responses such as dive ratios (dive duration in relation to post-dive recovery time) with respect to extrinsic factors such as prey depth or distribution (Gilmour et al., 2005). The prediction of an increase in post-dive recovery times (response variable) with increasing dive duration (predictor) is directly derived from the need to maintain long-term oxygen balance. For dives exceeding the ADL a disproportionate increase in such recovery times is predicted to accommodate less efficient (partially) anaerobic metabolism. Analyses of dive records for most species consistently yield highly significant positive correlations between dive durations and recovery times, largely as a result of the application of conventional measures of central tendencies to very large data sets (>>1000 dives, i.e., Figure 1). Such analyses account for little of the observed variation and violate parametric assumptions of homogeneity of variance. The few extant attempts at deriving the ADL from recovery time inflection estimates applied qualitative visual determination (Cook et al., 2008) subject to much interpretation. For example, applying a “bottom contour plot” visual approach to the data set shown in Figure 1 yields four inflection points at 1.92, 2.75, 3.5 and 3.67 min. However, these inflection points may be defined by single dives for which recovery times may be uncoupled from any immediate need to process any accumulated lactate if that is used as substrate in subsequent dives or accumulated over several dives. Furthermore, extremely large variance in recovery times for given dive durations tend to blur any effects and yield broad uncertainties in ADL estimates (i.e., Walton et al., 1998; Cook et al., 2008).

This lack of a consistently quantifiable behavioral manifestation of the ADL may be a result of several phenomena: (1) Individuals could choose to carry or accrue oxygen debts over several dives, only to pay off incurred debts after several dives. This would mean that any increase in recovery times resulting from lactate production could occur at a later time, not immediately following a dive exceeding the ADL. If accrual and payoff are flexible, our ability to analyze their association may be compromised. (2) The oxygen balance constraint is unidirectional in imposing minimum recovery times, while extended recovery times may be less constrained, for example by forces acting on foraging trip efficiency or individual fitness on much longer temporal scales, if at all. In this case, extended recovery times may appear “random” and could be influenced by variables not monitored by classic experimental designs, resulting in insufficient explanation of residual variation.

### Constraint lines

Constraint lines are best described as the boundaries that delimit point clouds in bivariate scattergrams. In a seminal paper, Thomson et al. (1996) highlighted that fundamental processes often constrain patterns of statistical variation. In the editor's note Carol Augspurger added that “Bivariate scattergrams convey ecological information not only when they show linear or curvilinear relationships, but also when they fall into diffuse clouds, as long as the clouds have informative edges.” These informative edges are called constraint lines, under the assumption that they represent the effect of limiting factors on a response variable with a lesser degree of unexplained residual variation than in the core of a diffuse cloud. Constraint lines have been applied in macro-ecology to describe and quantify the effects of limiting factors on response variables (Thomson et al., 1996; Guo et al., 1998; Kelt and Van Vuren, 2001). More recently, Anderson and Jetz (2005) used multiple constraint lines to characterize the field metabolic rate of endotherms within a broad latitudinal cline of environmental conditions. Their “metabolic constraint space” combined intrinsic and extrinsic factors to link the behavior and physiology of individuals to ecology and biogeography at a global scale. However, constraint lines have not been used to analyze intrinsic traits and the performance of individual animals. Here, I propose to use constraint lines—a technique that has gained increasing attention in the ecological community—to characterize physiological limits on performance, and specifically the ADL of diving, air-breathing vertebrates.

## Materials and methods

I examined existing data on dive behavior from Galápagos fur seals (*Arctocephalus galápagoensis*) obtained in 1990 and 1991 at the Cabo Hammond rookery on Fernandina Island, Galápagos, Ecuador, as previously described (Horning and Trillmich, 1997a). The original research was conducted under a research permit issued by the Galápagos National Park Service, and followed the “Guide for the Care and Use of Laboratory Animals” (Institute of Laboratory Animal Resources, 1996). In brief, Wildlife Computers Mk4 Timed-Depth Recorders (0–250 m × 1 m resolution, sampling at 5 s—except for two records sampled at 10 s) were deployed on 96 fur seals ranging in age from 6 months to adults. 194,361 dives were recorded during 1199 observation days. Mean deployment duration was 12.5 days (range 3–25 d). Depth records were processed as previously described (Horning and Trillmich, 1997a) to provide baseline-drift corrected sequential listings of dive durations and post-dive surface times.

### Dive data analysis

**Figure 2:**
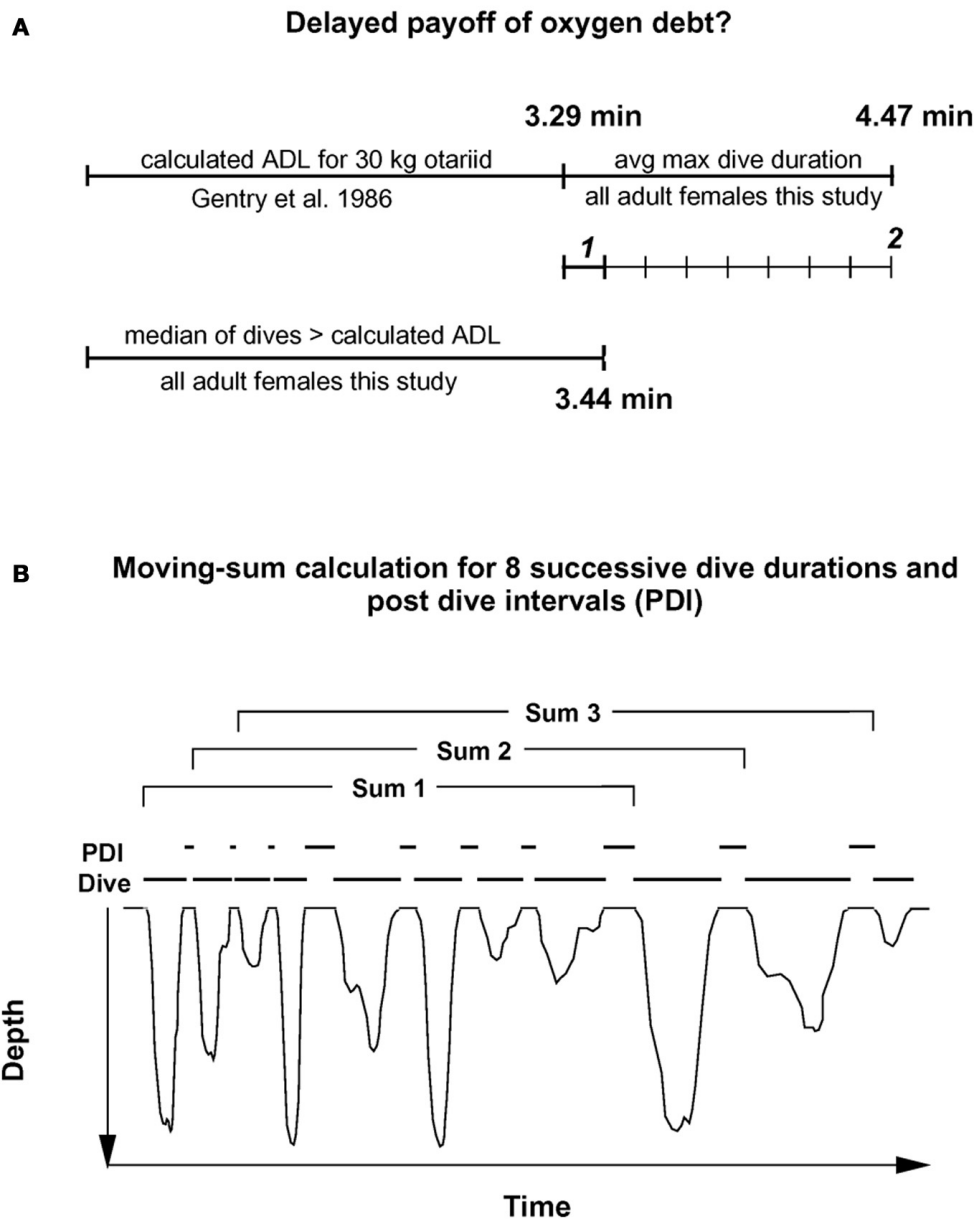
**Moving sum compensation of oxygen debt accrual. (A)** Estimating the number of dives after which the maximum anaerobic increment (see text) has been accrued. ***1*** represents the median anaerobic increment by a typical dive exceeding the calculated ADL. ***2*** represents the maximum anaerobic increment based on the difference between calculated ADL, and mean maximum dive duration. **(B)** The moving sum calculation. Dive durations for eight sequential dives are summed, and the corresponding eight post-dive recovery intervals (PDI) are summed (see text for details). For each successive sum, one new dive—PDI pair is added, and the oldest pair is dropped. This moving sum is performed for all dives recorded for a particular animal. Resulting plots are shown in Figures 3, 4, and 5.

To consider a hypothetical disproportionate increase in recovery times for dives exceeding the ADL, I first plotted post-dive recovery times against all dive durations for each individual, as shown in the example of Figure 1. While some increase in recovery times for longer dive durations was observed, no simple and obvious determination of the ADL was possible for any of the records. To compensate for a possible delayed payoff of an oxygen debt incurred during a (partially) anaerobic dive, I estimated the number of dives exceeding the ADL after which an accumulated, incremental oxygen debt would have to be dealt with for adult females as illustrated in Figure 2A. In a first step, I estimated the likely contribution to oxygen debt accrual by only those dives that exceed the calculated ADL. Gentry et al. calculated the ADL of a hypothetical otariid from oxygen stores and utilization rates by pooling available data from multiple otariid species. For a body mass range of 30–100 kg, their estimates were best described by the equation ADL = 1.57 × body mass^0.22^ (Gentry et al., 1986). The mean body mass of all 32 adult fur seal females in the data set was 28.8 kg (± 3.1 SD).

Using the above equation from Gentry et al., I estimated an ADL of 3.29 min for a 29 kg adult Galápagos fur seal female. The median duration of only those dives exceeding this estimated ADL was 3.44 min.

**Figure 3:**
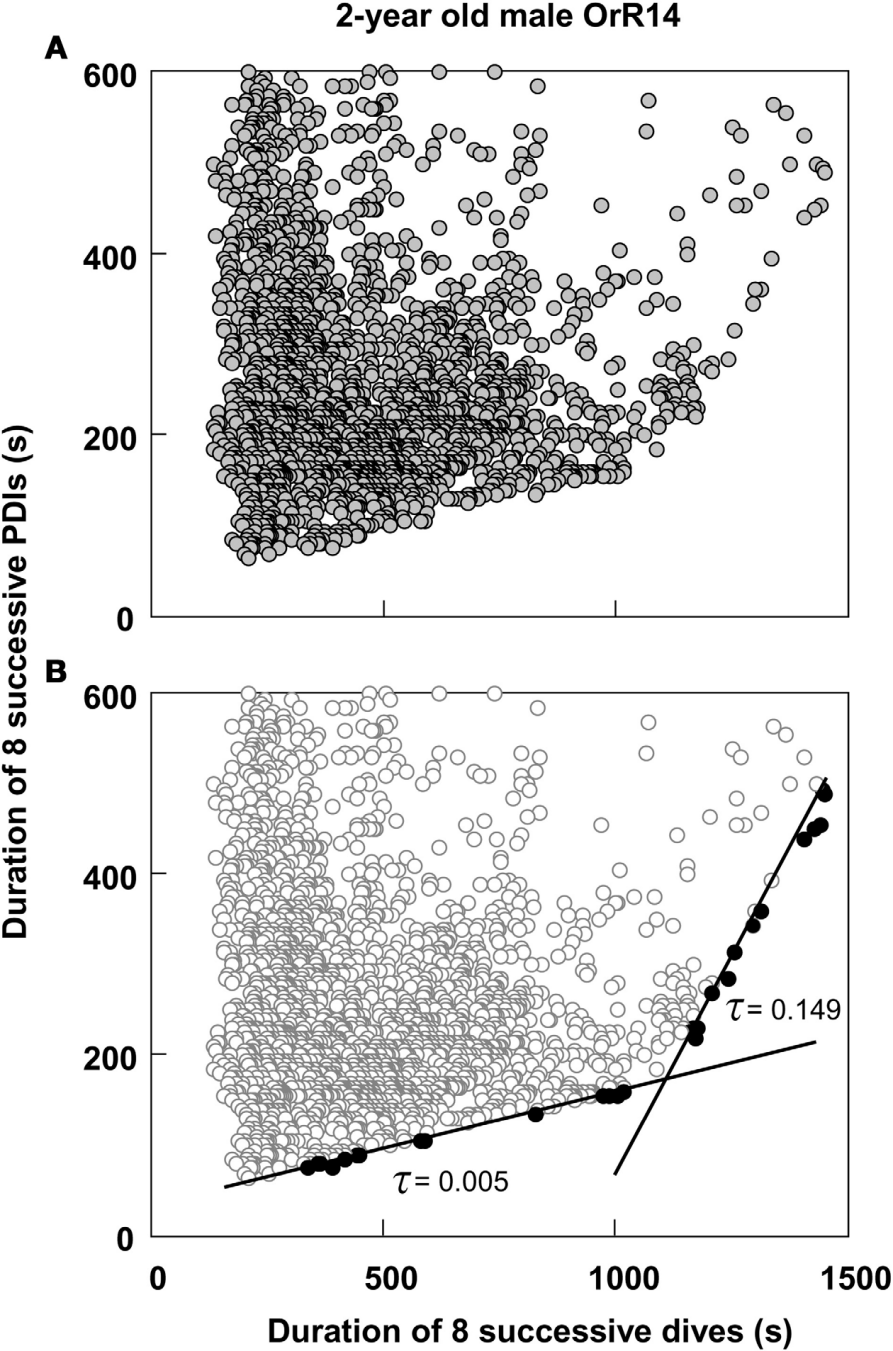
**Moving sum plot for 2-year old male Galápagos fur seal OrR14. (A)** The plot resulting from the moving sum calculation for eight successive dive—PDI pairs as explained in Figure 2B, for 2-year old male fur seal OrR14. **(B)** The best split Kendall-Theil quantile regressions yielding *n* ≤ 20 for the τ-th proportion (solid circles, *n* = 13 for each regression) with the lowest combined root mean square errors (see “Materials and Methods”). Dividing the abscissal value of the regression intersect by eight yields the equivalent duration for a single dive. The intersect at 1100 s corresponds to a single dive duration of 137.5 s or 2.29 min. This value is shown as a solid black triangle in Figure 1.

Thus, most dives by adult females in this study that exceeded the calculated ADL did so by only 0.15 min on average, or about 4.5%. This short apneustic duration in excess of the calculated ADL could be called the median anaerobic increment. Given that such a short anaerobic increment increases the oxygen debt only by a small amount, it is possible that such debt is accrued and payoff (in the form of increased recovery times) is deferred. Next, I estimated the possible number of typical anaerobic increments in a series of dives after which payoff can no longer be deferred. The maximum dive duration observed in each of these animals represents a rare performance extreme and as such an overall physiological limit which apparently cannot easily be exceeded. The average maximum dive duration of all 32 adult females in the study was 4.47 min, suggesting that the maximum anaerobic increment is 1.18 min (4.47–3.29). Under this assumption, after eight typical dives exceeding the estimated ADL by the median anaerobic increment, the maximum anaerobic increment is reached and payoff can no longer be deferred (Figure 2A). Thus, after eight of the most common, presumably partially anaerobic dives an increase in recovery times can no longer be avoided. To relate recovery times to dive durations under this scenario of multiple shorter anaerobic increments with payoff deferred over up to eight dives, the combined recovery times for eight sequential dives has to be related to the combined duration of eight sequential dives. To that extent, I calculated the moving-sum of all possible sequential combinations of eight successive Post-Dive-Intervals (PDI) and the corresponding sum of eight successive dives for all dives recorded for a particular animal, as illustrated in Figure 2B. The scatterplots resulting from the moving-sum transformation show all possible pairings of dive durations with recovery times for series of eight dives, including those with shortest observed recoveries. In the resulting moving-sum plots, linear increases in minimum post-dive recovery times for sets of eight sequential dives were observable as lower distribution boundaries in many of the dive records, as shown in Figure 3A. Beyond a distinct surface time inflection point (STI), minimum observed post dive surface recovery times increased at a disproportionate rate, as characterized by a second boundary of steeper slope. If larger or smaller integration values are used for the moving sum, the inflection point becomes progressively less pronounced.

### Determination of surface time inflection points

I used quantile regression (Cade et al., 1999) to separately quantify the lower boundaries for all combinations of two split segments (Yaeger and Ultsch, 1989; Granato, 2006) of all moving-sum data pairs for individual dive records, programmed in Turbo-Pascal (Borland). Since the original dive duration versus PDI data and the corresponding moving-sum data were strongly heteroscedastic (i.e., Figures 1 and 3A), I used the non-parametric Kendall-Theil robust line estimator (Sen, 1968; Granato, 2006) to calculate the τ-th regression quantiles for heterogeneous distributions where the τ-th proportion of data located below the regression line is weighted by 1-τ, and the 1-τ th proportion located above the regression line is weighted by τ (Cade et al., 1999). Calculations were simplified by using only cross-median paired slopes to determine the Kendall-Theil estimator (De Muth, 2006). Since individual dive records covered a broad range of dives from 511 to 6727 dives and were typically heavily skewed toward shorter duration dives and PDIs, I progressively decreased τ from an initial value of 0.5 in steps of 0.01 until the τ-th proportion contained between 5 and 20 samples, and while successive slopes or elevations differed significantly. I determined the significance of slope differences via a rank-sum test of slopes and elevations used to calculate the Kendall-Theil medians for α = 0.05. I performed this procedure iteratively for all combinations of split segments for values from 25 to 95% of the abscissal range (see Yaeger and Ultsch, 1989) and calculated the STI point as the intersection between the two quantile regression lines that yielded the lowest combined root mean square error (Granato, 2006), as illustrated in Figure 3B.

Since a split, two-segmented linear model always provides an equal or better fit (equal or lower combined root mean square error) than a single fit (Yaeger and Ultsch, 1989), I established two criteria to qualify an STI as reflecting a “disproportionate increase” in minimal post-dive recovery times: (1) the slope of the fit to the right of the STI has to be significantly greater than that to the left; (2) the STI has to meet a continuity restriction: the abscissa of the intercept has to fall within 10% of the split between segments. Dividing the STI value by eight yielded equivalent durations for single dives, beyond which minimal recovery times increased at a disproportionate rate.

## Results

**Figure 4:**
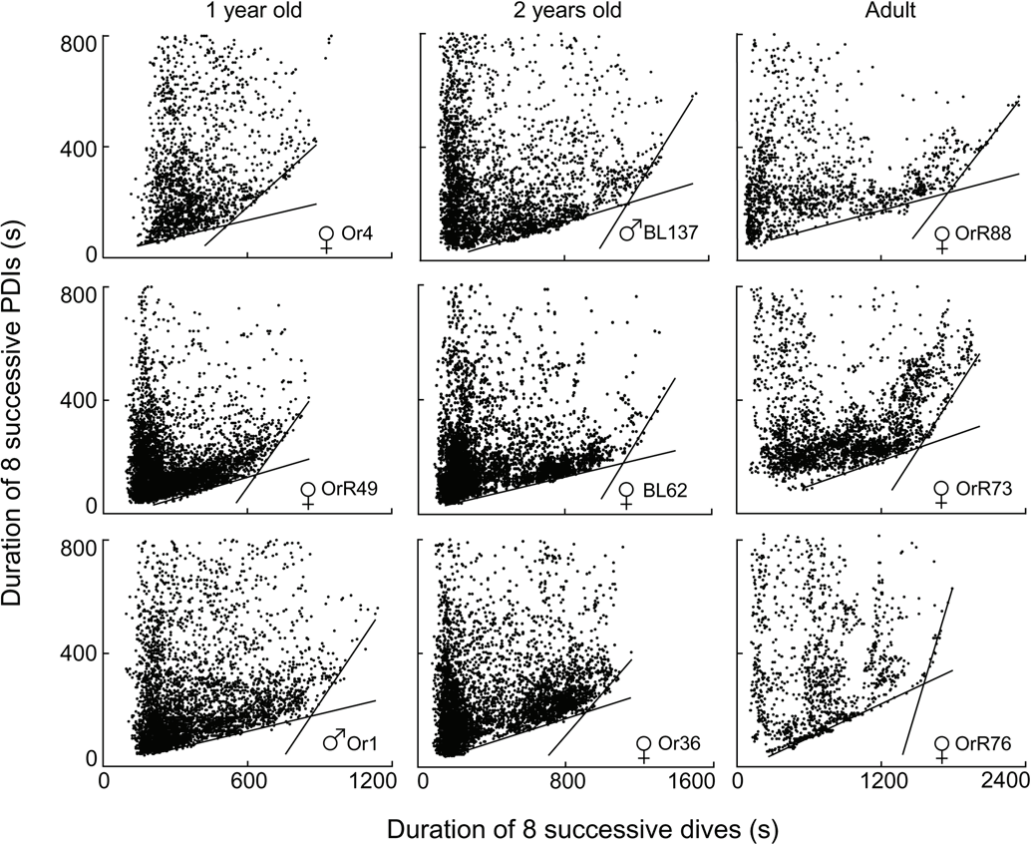
**Best split τ-th regression quantiles for nine different Galápagos fur seals of three different age classes.** Dividing the abscissal values of the intersection of the two regression lines by eight yields the equivalent durations for single dives.

**Figure 5:**
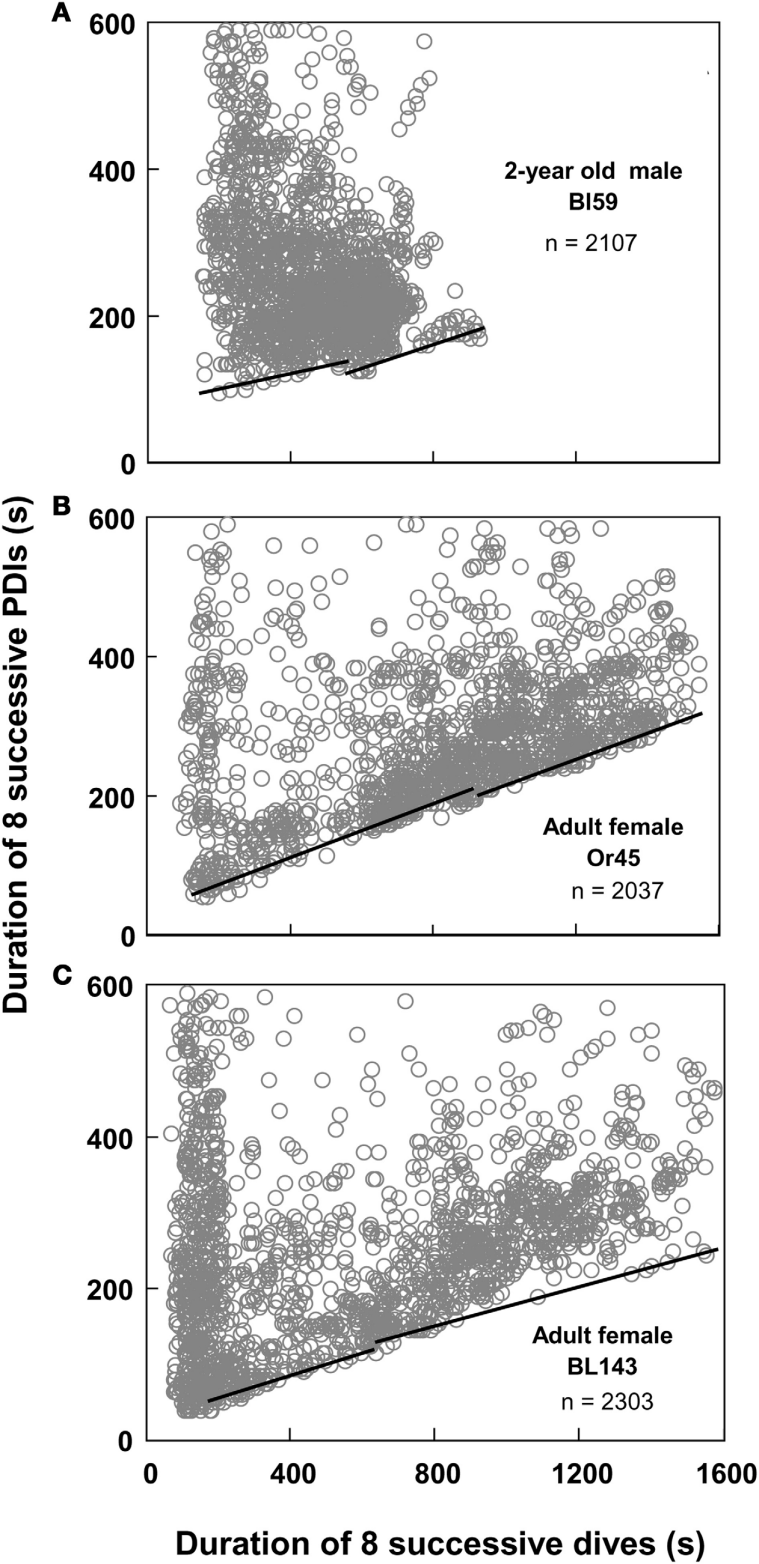
**Continuity and slope violations. (A)** Best split τ-th regression quantiles with a continuity violation: the abscissal value of the intersection between the two regression lines does not fall within 10% of the split. **(B,C)** Best split τ-th regression quantiles with a non-significant slope difference. Dividing the abscissal values of the regression intersects by eight yields the equivalent durations for single dives.

Of the 96 depth records examined, I excluded two records from subsequent analyses because of differing sampling rates (10″ versus 5″), and 12 records from ages <220 days since animals in this age class play in the water without diving and do not yet forage independently (Horning and Trillmich, 1997a). Of the remaining 82 records, three contained fewer than 400 dives (60, 167, and 371 dives respectively) and exhibited no apparent edges to the diffuse point clouds. 79 records allowed the computation of segmented regression quantiles. 51 of these records yielded two constraint lines with distinct STIs (i.e., Figure 4). Twenty-eight records violated previously set criteria of continuity (i.e., Figure 5A) or slope differences (i.e., Figures 5B,C). Records with slope violations exhibited a single lower constraint line with few or no points beyond the upper line limit (*n* = 24). These animals likely did not dive for durations beyond the STI. Records with a continuity violation exhibited few points or diffuse point clouds beyond the upper limit of the lower constraint line (*n* = 4). These animals did perform some dives beyond the STI, but were apparently spending more time at the surface than minimally necessary for such dives. For these records, I used the largest value on the abscissa for the lower regression (continuity violation) or for a single, combined regression (slope difference violation) to characterize the maximum observed dive duration *not* resulting in increased PDI.

## Discussion

The moving sum calculation uncouples the distribution boundaries from small scale (dive-by-dive) deviations from homeostasis enabled by the plasticity of the dive response. The resulting constraint lines yield consistent intercepts that are defined by a much larger number of dives than, and distinct from visually placed inflections in “bottom contour plots” (see Figure 1). The efficacy of the moving-sum plots in visualizing distinct and disproportionate increases in minimum observed recovery times for sets of dives does suggest that (1) Galápagos fur seals do at times exceed their ADL, (2) apparently do so by comparably small amounts on individual dives, and (3) may incrementally accrue an oxygen debt which is “paid off” when a threshold is reached after a series of contributing dives. The linear increase in minimum recovery times for dives sets < the STI (Figure 4) is contrary to earlier hypotheses on “optimal breathing” that suggest an asymptotic decrease in oxygen transfer rates with increasing PDI (Kramer, 1988). However, estimates of blood flow and PO_2_ measurements on diving seals (see Ponganis et al., 2011) and models of oxygen transport between tissues (Davis and Kanatous, 1999) suggest such a decrease as highly unlikely for surface intervals typically much shorter than 30 s for Galápagos fur seals.

Constraint-related data collected under steady-state, controlled laboratory conditions can often be adequately described by measures of central tendencies, but field data may rarely be, since the introduction of other factors that are less-, or not at all limiting tend to blur the point cloud in a skewed direction. Quantile regression appears a usable technique for the characterization of the lower boundary line resulting from the moving-sum transformation, and it avoids violating parametric assumptions that are challenging for large data sets typical in diving animal telemetry. This approach is particularly applicable under the a priori assumption of a functional, mechanistic relationship unilaterally constraining a specific response variable irrespective of its central tendencies influenced by many other variables that are rarely recorded, even if the predictor contributes only a small amount to the overall variance observed. As we move from the center of a distribution toward a constrained boundary, the contribution to variance shifts from unmeasured factors toward the measured predictor (Cade and Noon, 2003).

**Figure 6:**
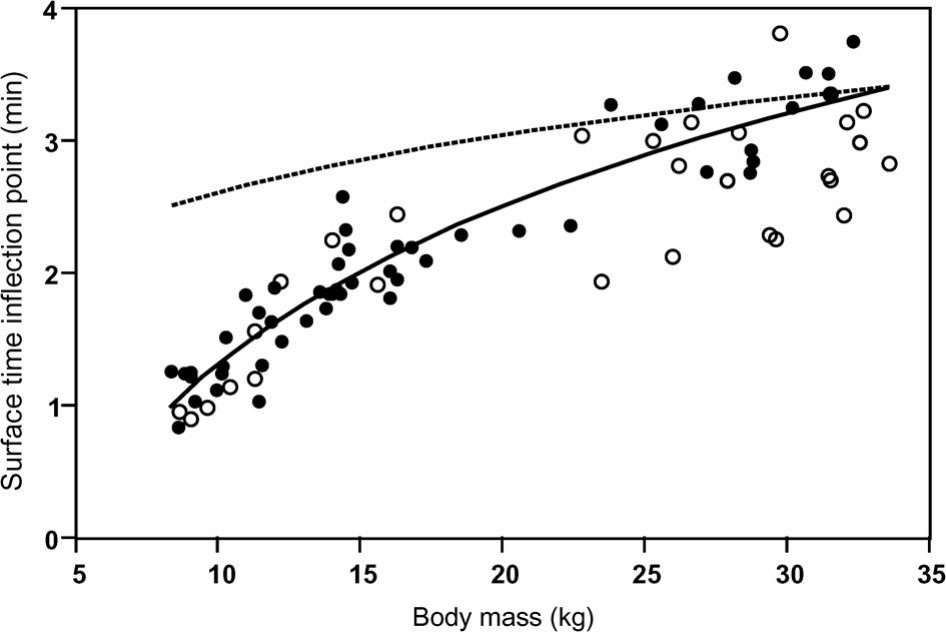
**The Surface Time Inflection point (STI) as a function of body mass.** The Surface Time Inflection points computed for single-dive durations from the intersections of best split quantile regressions are plotted against body mass (solid circles). The points are best described by the equation (solid line): *STI* = −2.76 + 1.77 × ln(body mass). The ADL for a hypothetical otariid calculated from body mass (calculated after Gentry et al., 1986 from the power equation ADL = 1.57 × body mass^0.22^) is shown (dashed line). Maximum observed dive durations ≤ STI for records with slope or continuity violations (see text and Figure 5) are shown as open circles.

**Figure 7:**
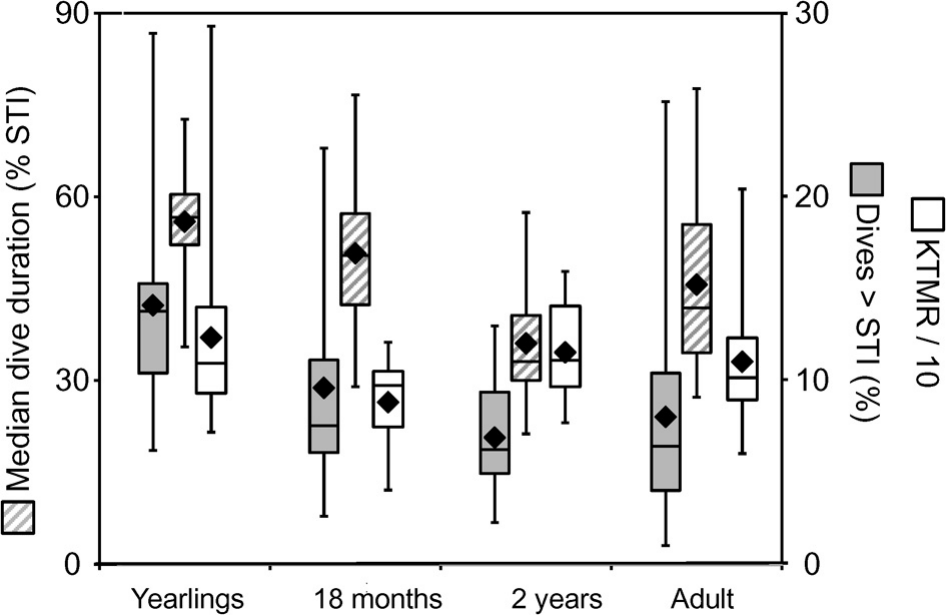
**Constraint metrics for juvenile Galápagos fur seals.** Three constraint metrics derived from the STI (see text) are shown for three juvenile age classes and adult female Galápagos fur seals. Yearlings and 2-year-old animals were observed during the cold season, 18-month-old juveniles were observed during the resource limited warm season, adult females during both seasons. Solid black rhombes are means, center box lines are medians, box limits and whiskers are inner and outer quartiles, respectively. The median dive duration of individuals is shown in percent of the STI (left y-axis). The percent of dives exceeding the STI and the Kendall-Theil Median Residual (KTMR, see text) are shown on the right y-axis.

Despite the postulated mechanistic relationship, my interpretation that the disproportionate increase in minimum surface times for dives exceeding the STI is driven by at least partially anaerobic metabolism, and thus is a manifestation of the ADL, has to be considered a hypothesis that remains to be tested. In the absence of empirical data on PO_2_ or blood lactate, we can presently only consider the physiological and ecological context to assess the validity of this hypothesis. As expected for the ADL, the STI does indeed exhibit a strong positive relationship to body mass (Figure 6). This relationship is best described by the equation STI (min) = a + b × ln [body mass(kg)] where *a* = −2.76, *b* = 1.77. Mean values (±S.D.) for age classes are: adult females 3.16 min (±0.37, *n* = 14), 1.5–2 years 1.98 min (±0.27, *n* = 23), yearlings 1.28 min (±0.23, *n* = 14). For 14 adult females the mean STI is only slightly below the calculated ADL of 3.28 min (calculated after Gentry et al., 1986 from the power equation ADL = 1.57 × body mass^0.22^). For younger animals with a lower body mass the STI falls increasingly below the calculated ADL (Figure 6). We previously showed that select parameters (blood hemoglobin concentration and hematocrit) related to oxygen storage and transport capacity of young fur seals reach levels of adult females by the age of one year (Horning and Trillmich, 1997b). However, muscle parameters such as muscle mass and myoglobin concentrations were not assessed and may mature at slower rates. Furthermore, Gentry and collaborators derived their equation describing the calculated ADL of a hypothetical otariid from parameter estimates combined from adults of multiple species (Gentry et al., 1986). Maximum and median dive performances (dive depths and durations, swim speeds, vertical travel distances per trip to sea) of younger fur seals ages 1–2 years range from 50 to 75% of values reached by adult females (Horning and Trillmich, 1997a), matching the pattern shown in Figure 6. The notion that juveniles are more constrained in their independent diving and foraging ability than adult females is further supported by their greater mass loss during lunar periods of reduced foraging efficiency resulting from periodically reduced prey accessibility (Horning and Trillmich, 1999). The percentage of dives exceeding the STI is only slightly and insignificantly higher for all juveniles combined (10.1%, ±5.7 S.D., *n* = 37) than adult females (7.99%, ±6.08 S.D., *n* = 14), but for yearlings (14.1%, ±5.4, *n* = 14) the difference to adult females is significant (Student's *t*, *p* < 0.012) (Figure 7). This likely explains the greater proportion of inflection points successfully determined in juvenile records (37 in 47) than adult females (14 in 32).

Thus, it appears that the performance envelope of the Galápagos fur seal is shaped by two constraint lines on either side of the STI. From the ADL interpretation of the STI, we can derive the expectation that *the proximity of recovery times to the “aerobic” boundary* for dives shorter than the STI should be influenced more by the intrinsic need to optimize foraging efficiency—for example when resources are scarce—rather than by limits imposed by physiological maturation, age or mass. This leads to the prediction that the Kendall-Theil Median Residual (KTMR) of the aerobic portion of the quantile regression should be lower during the equatorial warm season (February–April) when cold-water upwelling is suppressed, reducing nutrient influx and primary productivity (Barber and Chavez, 1983; Kogelschatz et al., 1985, and see Horning and Trillmich, 1997a) than during the more productive cold season (June–November). Indeed KTMR values dropped from a mean of 143.9 (±104.5 S.D.) for 20 yearlings observed during the cold season to a mean of 87.8 (±24.9 S.D.) for ten 18-month-old animals observed during the warm season (the difference was not significant with Student's *p* = 0.117) and increased again to 114.7 (±26.3 S.D.) for seventeen 2-year-old animals observed during the cold season (this second difference was significant with Student's *p* = 0.0186) (Figure 7). For adult females the effect was reversed, with mean KTMR values of 100.6 (±28.4) for 20 females observed during the cold season, and 124.6 (±32.5) for 12 females observed during the warm season (*p* = 0.042). However, this is likely driven by a much greater energetic demand on lactating females during the peak of the (cold) breeding season, when they are more likely to have a dependent pup unable to feed itself, possibly in addition to an older still dependent yearling.

From the ADL interpretation of the STI we can also derive the notion that *the proximity of typical dive durations to the ADL* should be indicative of the extent to which individuals exploit the full extent of behavioral plasticity as constrained by physiological maturation and body mass. This leads to the specific prediction that the median dive duration expressed as a percentage of the STI should be highest for yearlings, and decline for older juveniles following weaning, followed by an increase for adult females. Indeed, this value declines from a mean of 55.9 (±10.1) for fourteen yearlings past 50.8 (±14.2) for seven 18-month-olds (n.s. *p* = 0.37) to 36.1 (±9.6) for nineteen 2-year old animals (*p* = 0.012) (Figure 7), and then increases again to 45.66 (±14.8) for fourteen adult females (*p* = 0.0495).

The variance observed in these three measures of individual constraint (KTMR, the median dive duration expressed in percent of STI, and the percent of dives exceeding the STI) in the Galápagos fur seals is fully consistent with our prior findings of constraints across a range of temporal and spatial scales (Horning and Trillmich, 1999). Adult females appear most challenged to optimize diving behavior during the (cold) reproductive season, whereas juvenile fur seals appear most challenged during the warm, resource limited season, and after weaning. As expected, younger juveniles are more constrained and need to push their limits to a greater extent than older juveniles. Adult females however appear to be additionally constrained by the energetic demands of lactation and are diving closer to their physiological limits than 2-year old juveniles.

Irrespective of an empirical validation of the STI = ADL hypothesis, I propose that these three measures of the proximity of central tendencies to the constraint boundaries in the dive record scatterplots may be useful metrics to quantify the need to optimize foraging behavior, and how close to the limits of their plasticity diving animals may operate at several temporal scales.

### Conflict of interest statement

The author declares that the research was conducted in the absence of any commercial or financial relationships that could be construed as a potential conflict of interest.
